# Sound Practice, Sound Philosophy

**Published:** 1873-04

**Authors:** M. S. Dean


### ARTICLE III.

### *Sound Practice, Sound Philosophy.*—Continued.

BY M. S. DEAN, D. D. S.

One word more in regard to the false philosophy of another class of advocates of smooth points, and I will leave this portion of the subject to you. What I would now say would have come in more naturally, perhaps, in that portion of the paper where allusion was made to the practice of drawing the gold over the margins, by beating the gold with the mallet, but this matters little. The point is this: It has been contended that the thin walls—walls entirely composed of enamel, can be filled more completely and safely with the smooth than the serrated points. This is not a new nor very strange assumption, since the advocates of this style of instruments claim for them everything that is desirable in the accomplishment of successful results. But the *manner* in which this is accomplished, is what I wish to direct your attention to. It is not done in the usual way by packing the gold against the side of the frail walls, but by utilizing the ductile properties of the metal, and by the mallet force upon the face of the gold plug, drawing it snugly and perfectly against the walls and margins of the cavity. This is done on the same principle that a cubic slug is made to adjust itself to the sides of a gun barrel, when it is vigorously rammed down with a steel ramrod. I have seen this practice taught in some of our journals, but I am unable to refer to the article, except from memory, as they all took to themselves wings on the 9th of October last. "Though lost to sight, to memory dear" —very dier!

If the enamel was as strong as fine steel, this might be sound philsosphy, provided our patients were made of steel also.

Now I do not contend that such false theories, in regard to filling with smooth points, are the accepted doctrine of the entire class who advocate their use, but they are the theories of those who would be prominently known as the leaders in this practice. The more philosophical use points properly adapted, and in shape resembling Varney's. With these, I have no doubt that a few careful and skillful practitioners are able, in *favorable* cases, to put in fillings as perfect as can be made in any other way. But I do contend that in the most favorable case for smooth points, work can be done equally well with properly serrated ones, and with less trouble; and, also, that in a vast majority of cases, cavities may be filled with more ease, and perfectly, with the serrated instruments.

It may be good philosophy to change from one style of instrument to another, and one *form* of gold to another occasionally, even if the newly adopted ones are not, *in themselves*, as good as the rejected ones.

The mind constantly fixed for a long period of time upon the operation of filling teeth with one form of instrument and one form of gold, finds in a change of this kind interest and relaxation. The routine of labor is relieved of its monotony. Hence, after the dentist has become fatigued by the long usage of instruments properly fitted for his purpose, he may be able to accomplish as good results with those which are less adapted to his work. So also, the mind finds relaxation in the change of gold; and the dentist may, perhaps, accomplish better results by not confining himself to either the light or heavy Nos., nor to the soft or cohesive foils. In my own case I find that my mind is better adapted to using the heavy Nos. one day, and perhaps the very next day the lighter Nos. seem more congenial to my mental condition. And so I find it with other operators. I therefore conclude that it is sound philosophy and sound practice to change occasionally the style of instruments and the form of material used.

In the treatment of alveolar abscess our success depends in a great measure upon sound philosophy. The treatment of this disease is frequently—*generally* too often—repeated and too long continued. Some treat these cases as though the irritation of the therapeutic agent should be kept up as the irritation continues. We hear of those who treat very thoroughly, injecting creosote or its equivalent into the canal of the root every day or two at least, for months, or, in fact, until becoming discouraged, its further treatment is abandoned. When, shortly afterwards, the abscess recovers of itself!

One or two applications of creosote or carbolic acid are usually sufficient. The continued applications afterwards produce, instead of relieving, the trouble. The most difficult abscesses to treat are those of the lower bicuspid; for the reason that they do not usually find an opening through the alveolus, and because of the greater difficulty of removing *all* the *debris*. But if all the decayed or foreign matter be completely removed, this class of abscesses will yield as readily to treatment as those of the upper jaw.

An abscess with a fistulous opening is more easily treated than those having no such opening; for, in the former, the irritating particles of matter will be washed out, or find their way through the fistula. Hence, greater care must be taken to remove patiently and thoroughly all extraneous matter from the canals in roots having no fistulous opening, before therapeutic remedies are applied, and extreme caution exercised during this operation, to prevent irritating particles from being forced through the foramen.

There are, however, exceptional cases, in which it is necessary to make repeated applications before healthy action of the parts is restored. Yet, in such cases, a sufficient interval of time should be allowed between the applications, for otherwise we might prevent a speedy cure. No diseased tissue can become healthy, nor, if healthy, remain so if daily subjected to such treatment as is usually employed to cure an alveolar abscess.

It is neither sound philosophy nor sound practice to persist in the endeavor to restore to health the nerve pulp, in cases where the tooth has been filled for months and the pulp has been more or less painful during that time, and has become highly sensitive to warm drinks, and at times almost intolerable.

But the case is different when the pulp is exposed and has room for expansion. Here the entire pulp may not be involved, or much diseased, and may be amenable to treatment. But in the former case the tissues have suffered more severely, aud are in a partial state of disorganization, the whole mass being *in articulo mortis*, and can be saved only by a miracle. To treat such a tooth by applying external remedies, or to remove the filling and undertake to restore the nerve pulp to health, would be only to prolong the agonies of our patron.

The most skillful and conservative surgeons must sometimes amputate a limb to avoid more serious results, and so must the most conservative dentist, for a similar though less potent reason, occasionally in practice destroy the nerve pulp.

Now, gentlemen, a word in regard to the restoration of the contour of the teeth, and I will leave the unfinished subject to you. I consider the universal practice of restoring the contour of the teeth unsound. In many cases, it is true, it is very important to be done, being essential to the mastication of food as well as the distinct articulation of the voice, and in other cases it may add to the appearance of the teeth. But in the opinion of the writer this operation is performed many times when it would have been better practice to have made a less artistic operation. It is impossible in many cases, where a partial portion of the crown of a molar tooth is to be restored, to anchor the filling sufficiently strong to withstand the force applied by the antagonizing molar. And more especially where the force is in such a direction as to test the strength of the retaining points. The great force almost continually applied would tear away, after a few years' strain, the best possible retainers.

In the bicuspids too, there are many cases in which it is not the best practice to restore fully the contour of the tooth.

Take for example the lower bicuspid—very large posterior cavity—the pulp not exposed but so nearly so that it is prudent to protect it with ox. chloride of zinc. After the cavity is prepared, the labial and buccal walls are very thin, though they have been cut away extensively with the file, and the anterior wall, likewise, has but a slight dentinal support. Now, it will not be a difficult operation to restore its original contour, and the temptation to do so, as the work progresses, is strong.

We might accomplish this in such a manner that it would withstand the strain of mastication for a few years—and but a few. The labial and buccal walls were too weak to allow grooves to be cut in them—and to attempt a retaining pit in the cervical wall (or posterior base) hazardous, and must be small—any other than a small pit in the anterior port of the cavity impossible.

In such cases there can be no doubt that it is better practice and sounder philosophy to make the filling an inclined plane, so that the force of mastication will be in a direction to retain, rather than to dislodge it.

I should almost invariably favor contour fillings in the incisors and cuspids—at least whenever the filling be securely anchored, as it renders the tooth more serviceable—and the articulation of both the tooth and voice more natural.

